# Biological and Analytical Stability of a Peripheral Blood Gene Expression Score for Obstructive Coronary Artery Disease in the PREDICT and COMPASS Studies

**DOI:** 10.1007/s12265-014-9583-3

**Published:** 2014-08-14

**Authors:** Susan E. Daniels, Philip Beineke, Brian Rhees, John A. McPherson, William E. Kraus, Gregory S. Thomas, Steven Rosenberg

**Affiliations:** 1 CardioDx, Inc., 600 Saginaw Drive, Redwood City, CA USA; 2 Vanderbilt University, Nashville, TN USA; 3 Duke University School of Medicine, Durham, NC USA; 4 Long Beach Memorial, Long Beach, CA USA; 5 University of California, Irvine, CA USA

**Keywords:** Gene expression, Genomics, Atherosclerosis, Coronary artery disease

## Abstract

A gene expression score (GES) for obstructive coronary artery disease (CAD) has been validated in two multicenter studies. Receiver-operating characteristics (ROC) analysis of the GES on an expanded Personalized Risk Evaluation and Diagnosis in the Coronary Tree (PREDICT) cohort (NCT no. 00500617) with CAD defined by quantitative coronary angiography (QCA) or clinical reads yielded similar performance (area under the curve (AUC) = 0.70, *N* = 1,502) to the original validation cohort (AUC = 0.70, *N* = 526). Analysis of 138 non-Caucasian and 1,364 Caucasian patients showed very similar performance (AUCs = 0.72 vs. 0.70). To assess analytic stability, stored samples of the original validation cohort (*N* = 526) was re-tested after 5 years, and the mean score changed from 20.3 to 19.8 after 5 years (*N* = 501, 95 %). To assess patient scores over time, GES was determined on samples from 173 Coronary Obstruction Detection by Molecular Personalized Gene Expression (COMPASS) study (NCT no. 01117506) patients at approximately 1 year post-enrollment. Mean scores increased slightly from 15.9 to 17.3, corresponding to a 2.5 % increase in obstructive CAD likelihood. Changes in cardiovascular medications did not show a significant change in GES.

**Electronic supplementary material:**

The online version of this article (doi:10.1007/s12265-014-9583-3) contains supplementary material, which is available to authorized users.

## Introduction

The diagnosis of obstructive coronary artery disease (CAD) specifically, and atherosclerosis, more broadly, is a significant challenge. Although a variety of non-invasive testing are usually performed in patients with symptoms suggestive of CAD, including stress ECG, echocardiography, and myocardial perfusion imaging, less than half of patients without known CAD have significant disease upon invasive angiography [1]. Especially as the prevalence of significant CAD has decreased, improved risk stratification and more sensitive methods for detecting obstructive CAD are needed, to reduce the costs and morbidity associated with invasive testing [2].

Given the central role of a maladaptive inflammatory response in atherosclerosis and CAD development [3, 4], we analyzed peripheral blood gene expression in a series of microarray and real-time PCR (RT-PCR) studies, as a possible methodology for the sensitive, non-invasive, and non-radiation-utilizing detection of CAD [5]. These observations led to the development [6] of a gene expression score (GES) for obstructive CAD likelihood, comprising 23 gene expression levels, age, and sex, which is reported on a 1–40 scale, with subsequent validation in two multicenter studies [7, 8] (Personalized Risk Evaluation and Diagnosis in the Coronary Tree (PREDICT) and Coronary Obstruction Detection by Molecular Personalized Gene Expression (COMPASS)), in almost 1,000 non-diabetic patients referred for invasive angiography and myocardial perfusion imaging, respectively. In addition, the GES was shown to be significantly correlated with a composite endpoint of cardiovascular events and revascularizations [9].

Although the overall evidence for the utilization of gene expression testing for CAD diagnosis is promising [10], additional questions such as the reproducibility of the technology [11], dependence of test performance on ethnicity [12], and the schedule of testing have been raised. In this work, we describe testing of more than 1,500 patients from the PREDICT study, including those of various ethnicities, and the variation in the GES in serial testing over 1 year in 192 patients from the COMPASS study.

## Methods

### Study Populations

For the PREDICT (NCT no. 00500617) study, a total of 2,811 non-diabetic subjects were recruited. This entire study encompassed the gene discovery, development, and first validation study for the GES [6, 7], as well as post-validation cohorts. PREDICT enrolled symptomatic patients or high-risk asymptomatic subjects clinically referred for invasive coronary angiography, with no previously known history of CAD or revascularization. Key exclusion criteria included acute coronary syndromes, severe non-coronary cardiovascular disease, systemic infectious or inflammatory diseases, and the use of immunosuppressant or chemotherapeutic agents. For the current study, subjects enrolled after the completion of the discovery and development phases (*N* = 896) were included (*N* = 1,733). Post-validation recruitment was limited to non-diabetics and preferentially enrolled female subjects.

The second section of this work is based on an extension of the COMPASS (NCT no. 01117506) study population of 431 patients. The COMPASS study enrolled symptomatic non-diabetic patients with no history of MI or revascularization who were clinically referred for myocardial perfusion imaging [8], with similar exclusion criteria as for PREDICT. Patients in this study either had invasive angiograms, if clinically indicated, or research CT angiograms to determine coronary anatomy. The top four enrolling sites in this study were approached, IRB approval obtained, and patients at these sites consented for a second blood draw approximately 1 year after study entry.

### Acquisition of Angiograms and Core Laboratory Measurements

For PREDICT, analyses were performed on both the clinical invasive angiography reads, performed according to site protocols, as well as the quantitative coronary angiographic (QCA) reads, determined as described previously [7, 13]. Cases were defined as ≥70 % stenosis by clinical read or ≥50 % stenosis by QCA based on prior work [7]. For COMPASS, anatomical data at study entry were determined by QCA if invasive angiograms were performed or by core-laboratory CT angiography as described [14]. Core-laboratory measurements by two independent readers were utilized to define cases as ≥50 % stenosis [8].

### Measurement of Gene Expression Score

We utilized a previously validated quantitative RT-PCR (qRT-PCR)-based peripheral blood gene expression test run in a CLIA-certified laboratory (Corus® CAD; CardioDx, Inc; Palo Alto, CA, USA) [6, 7, 15], which is comprised of age, sex, and RNA levels of 23 genes expressed in peripheral blood cells. The test reports a score of 1–40, with higher scores associated with higher likelihood of obstructive CAD. Whole blood samples were collected in PAXgene® tubes (Pre-Analytix, Valencia, CA, USA) prior to myocardial perfusion imaging (MPI) or invasive angiography, treated according to the manufacturer’s instructions, then frozen at −20 °C. RNA purification, cDNA synthesis, and qRT-PCR were performed as previously described with the median values for all PCR reactions used for score calculation [6, 7, 15]. The GES was calculated from patient age, sex, and the median Cp values for the 23 genes as shown in the supplementary files [6, 7]. All GES measurements were run in 2013 on stored samples and where appropriate compared to the original values obtained in 2008 and reported previously [7].

### Statistical Analysis

Analyses were done in R Version 3.1.0 [16] and used the lm function for linear regression, the aov function for ANOVA, and the rcorr.cens function in the Hmisc library for receiver-operating characteristics (ROC) analysis. Continuous variables were expressed as mean ± SD or as median (inter-quartile range), as appropriate based on the distribution. Continuous variables were compared by *t* test (two-tailed) and categorical variables using the chi-squared test.

## Results

**Fig. 1:**
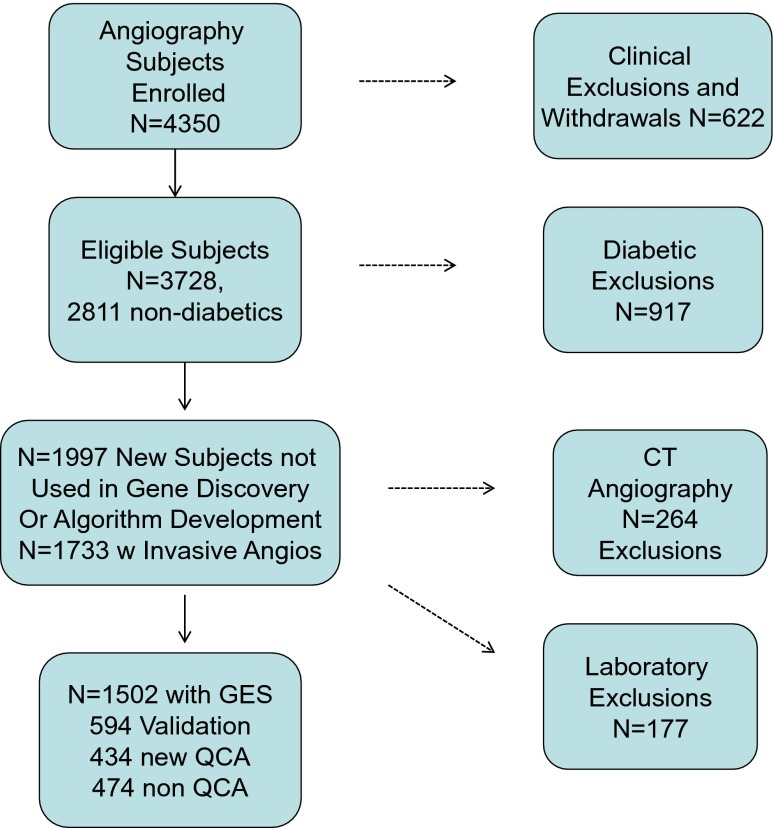
Patient flow for the PREDICT study cohorts. A total of 3,728 patients who met the original inclusion criteria were enrolled, comprising 2,811 non-diabetic and 911 diabetic subjects, with only the former as candidates for the current studies. Those non-diabetic subjects involved in previous discovery and development efforts (*N* = 814) as well as 264 who did not have invasive angiograms were excluded, yielding 1,733 subjects with clinical angiographic reads. There were 177 laboratory exclusions, resulting in 1,556 (90 %) which yielded valid GES measurements. For the QCA subset, a total of 1,082 patients were tested and the final set comprised 1,028 patients (95 %, 54 did not pass GES QC). There were an additional 474 subjects with clinical angiographic reads and GES yielding the final clinical cohort of 1,502

The PREDICT study enrolled a total of 3,728 subjects of whom 2,811 were non-diabetics, divided into sequential cohorts for gene discovery, algorithm development, validation, and post-validation studies, and stratified by the availability of clinical versus QCA angiographic data (Fig. 1). For this work, a total of 1,502 patients were analyzed for whom clinical and demographic data, invasive angiography, and GES were obtained. In addition, subsets corresponding to the original validation set (*N* = 648), and those for whom QCA had been performed (*N* = 1,038), were analyzed. Clinical and demographic data for these cohorts are detailed in Tables 1 (validation set) and 2 (complete QCA and clinical sets).

**Table 1:** Demographic and clinical characteristics of PREDICT validation subjects

	Full set^a^	Original validation^b^	New validation^c^	Intersection^d^
*N*	648	526	594	501
Female	285 (44 %)	227 (43 %)	266 (45 %)	222 (44 %)
Age	60	60	60	60
Race—White-non-Hispanic	580 (90 %)	474 (90 %)	540 (91 %)	453 (90 %)
Chest pain				
Typical	263 (41 %)	213 (41 %)	246 (41 %)	207 (41 %)
Atypical	162 (25 %)	133 (25 %)	149 (25 %)	127 (25 %)
Non-cardiac	12 (2 %)	12 (2 %)	12 (2 %)	12 (2 %)
None	210 (33 %)	167 (32 %)	187 (32 %)	155 (31 %)
Hypertension	425 (66 %)	345 (66 %)	391 (66 %)	331 (66 %)
Dyslipidemia	426 (68 %)	341 (67 %)	395 (69 %)	324 (67 %)
BMI	30.8	30.7	30.7	30.8
Systolic BP	135.8	135.2	135.6	135.3
Diastolic BP	78.6	78.1	78.7	78.2
Smoking^e^	115 (18 %)	92 (18 %)	109 (18 %)	88 (18 %)
CAD by QCA^f^	238 (37 %)	192 (37 %)	219 (37 %)	184 (37 %)
CAD by clinical read^g^	195 (30 %)	161 (31 %)	183 (31 %)	154 (31 %)

^a^The complete validation set described in Elashoff et al. [6] without one patient who was a late clinical exclusion for diabetes

^b^Those patients whose samples passed all metrics for inclusion in the original validation study completed in 2008

^c^Patients from the full set who passed all GES metrics for inclusion for data completed in 2013

^d^The patients for whom GES was obtained in both 2008 and 2013

^e^Smoking refers to current smoking

^f^Patients were defined as cases with CAD using ≥50 % stenosis by QCA

^g^Patients were defined as cases with CAD using ≥70 % stenosis by clinical read

To determine sample and process stability, we measured GES for the validation set (*N* = 648) [7] from PAXgene® tubes stored at −20 ° C for approximately 5 years. In the original analysis, GES had been obtained for 526 subjects, whereas in the current iteration, a total of 594 passed GES QC, with an intersection of 501 subjects. Clinical and demographic data for these groups did not differ significantly with 56 % male, (average age 60 years), 90 % non-Hispanic Whites, 69 % symptomatic and 31 % asymptomatic presentation, 37 % obstructive CAD (≥50 % stenosis by QCA), and mean GES of 19.8 (Table 1). The average change in the GES between the original work performed in 2008 and the current testing performed in 2013 for this group (*N* = 501) was 0.53, on the 1–40 scale, corresponding to approximately a 1 % change in disease likelihood. There was no significant change in test performance by ROC analysis between the original and most recent data sets (area under the curve (AUC) = 0.70 for both, *N* = 501) (Table 3).

The complete QCA cohort (*N* = 1,028) included subsequent preferential enrollment of female subjects raising the percentage to 55 % overall but otherwise was similar with respect to demographic parameters (average age 60 years, 91 % non-Hispanic Whites, 71 % symptomatic, and 33 % obstructive disease by QCA (28 % by clinical read of ≥70 % stenosis)) with a mean GES of 18.3. The complete clinical cohort (*N* = 1,502), which includes the QCA cohort, was 54 % female and showed similar characteristics with 27 % obstructive disease by clinical read (Table 2).

**Table 2:** Clinical and demographic characteristics of PREDICT validation, QCA, and clinical populations

	New validation set^a^	Complete QCA set^b^	Complete clinical set^c^
*N*	594	1028	1502
Female	285 (44 %)	561 (55 %)	809 (54 %)
Age	59	60	60
Race (White–non-Hispanic)	540 (91 %)	936 (91 %)	1364 (91 %)
Chest Pain			
Typical	246 (41 %)	438 (43 %)	651 (43 %)
Atypical	149 (25 %)	276 (27 %)	401 (27 %)
Non-cardiac	12 (2 %)	19 (2 %)	32 (2 %)
None	187 (32 %)	294 (29 %)	415 (28 %)
Hypertension	391 (66 %)	681 (67 %)	978 (66 %)
Dyslipidemia	395 (69 %)	671 (68 %)	975 (67 %)
BMI	30.7	30.4	30.3
Systolic BP	136	135	135
Diastolic BP	79	78	79
Smoking	109 (18 %)	186 (18 %)	277 (19 %)
CAD by QCA	219 (37 %)	343 (33 %)	343 (33 %)
CAD by clinical read	183 (31 %)	291 (28 %)	410 (27 %)

^a^The complete set of PREDICT patients for whom GES results were obtained in the 2013 testing

^b^The entire number of patients for whom QCA and GES results were obtained, including those in the new validation set

^c^All the non-diabetic patients in the study (see Fig. 1) who had clinical invasive angiographic reads and GES, determined in 2013

ROC analysis for all data sets showed very similar results to the original validation study (Table 3) with no significant difference by sex or clinical versus QCA case-control definitions. Analysis of non-Hispanic Whites (*N* = 1,364) and other ethnicities (*N* = 138) showed significant and similar AUCs for both groups (Table 3).

**Table 3:** ROC analysis for obstructive CAD of all subject data sets^a^

Data set	*N*	AUC	Std error	*p* value
Original validation (2008)^b^	526	0.70	0.02	<0.001
Males—original validation	299	0.66	0.03	<0.001
Females—original validation	227	0.65	0.05	0.0015
New validation (2013)^c^	594	0.70	0.02	<0.001
Males—2013 validation	328	0.66	0.03	<0.001
Females—2013 validation	266	0.64	0.04	0.001
Common validation set^d^	501	0.70	0.02	<0.001
Total QCA population	1,038	0.70	0.02	<0.001
Clinical read on QCA population	1,038	0.68	0.02	<0.001
Clinical read entire population	1,502	0.70	0.02	<0.001
Males—clinical read—all	693	0.66	0.02	<0.001
Females—clinical read—all	809	0.64	0.03	<0.001
White—non-Hispanic—all	1,364	0.70	0.02	<0.001
Non-White—all^e^	138	0.72	0.06	0.0002

^a^For QCA, obstructive CAD was defined as ≥50 % stenosis; for clinical reads, the threshold was ≥70 %

^b^Patients from the *N* = 648 cohort for whom QCA and GES were obtained as reported in Rosenberg et al. [7]

^c^Patients from the *N* = 648 cohort for whom QCA and GES were obtained in 2013

^d^The intersection of the 526 and 594

^e^Represents 75 African-American, 38 Hispanic, and 25 others by self-reported ethnicity

**Fig 2:**
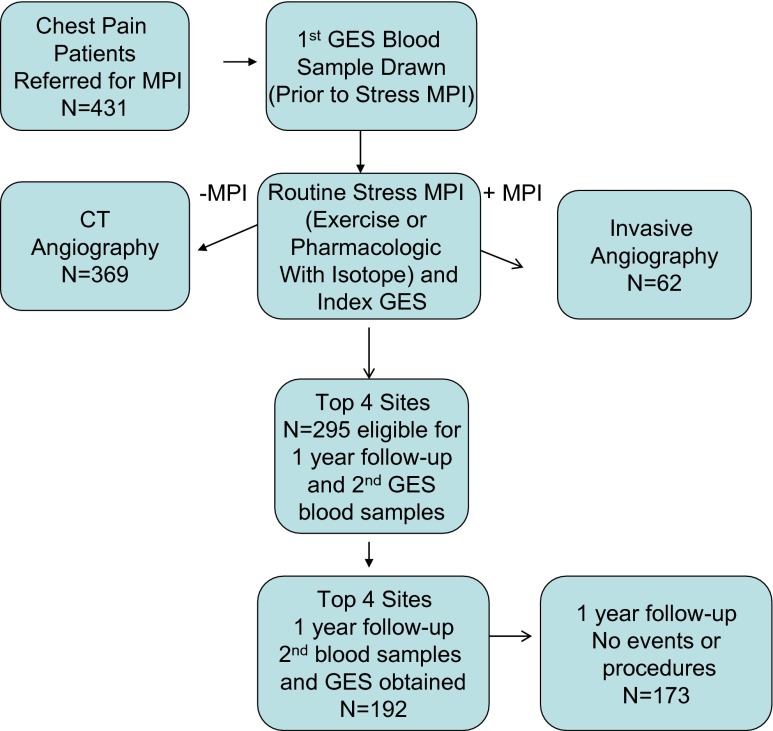
Patient enrollment and flow for COMPASS (NCT 01117506) study index and follow-up GES measurements. From the original 431 COMPASS subjects (all non-diabetic) with CT or invasive angiograms, GES, and MPI, the four highest enrolling sites enrolled 295. A total of 195 (66 %) consented and were enrolled for the second-draw study with GES being obtained on 192 (98 %); of these, 173 did not have events or procedures prior to the second GES measurement

**Fig 3:**
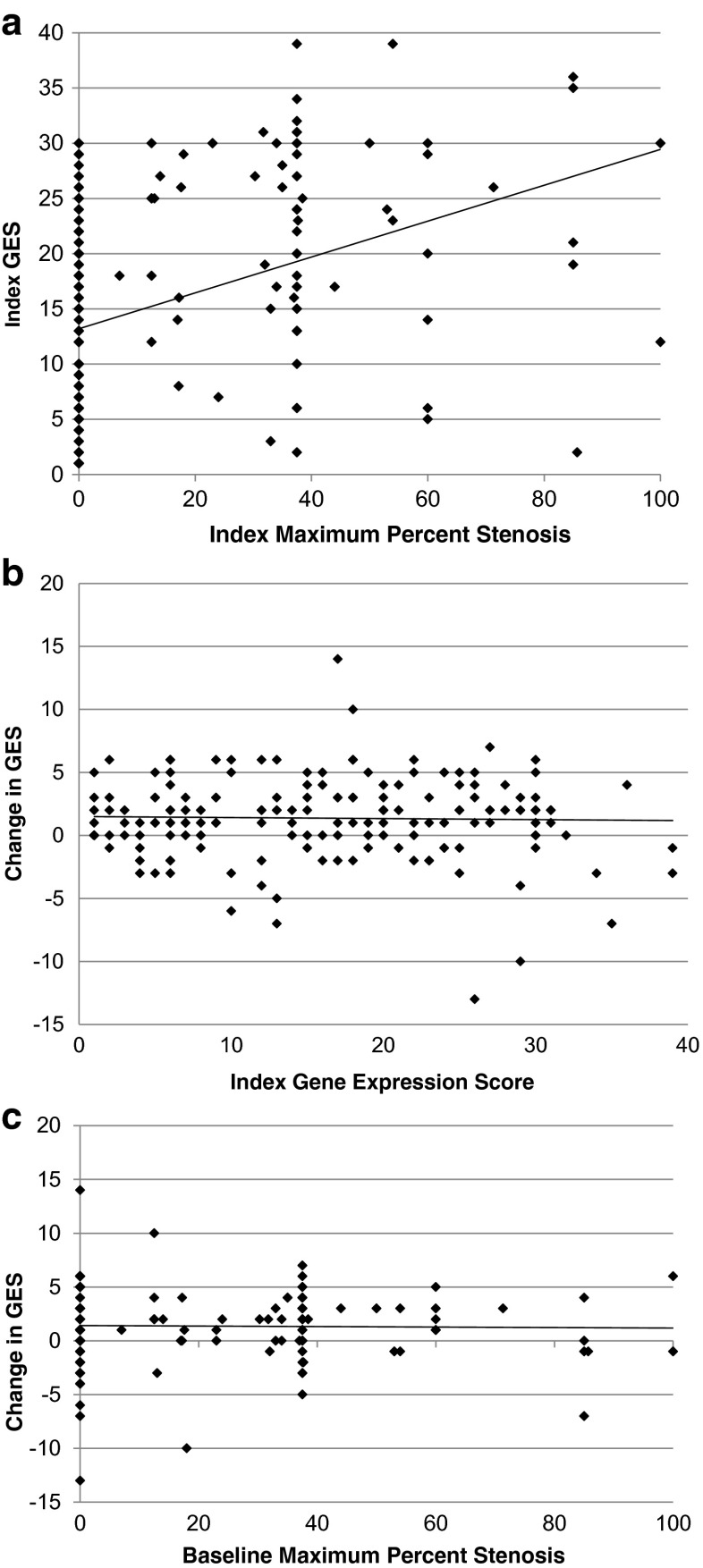
COMPASS analyses of index and second-draw GES. **a** Relationship between index GES and maximum percent stenosis determined by core-laboratory CTA or QCA for the 173 patients without events or procedures between GES measurements is shown. Core-laboratory maximum percent stenosis (MPS) was determined as described [8], in stenosis categories by two independent readers. The median of the category stenosis range is used in each case. The GES is significantly correlated with MPS (*r* = 0.39, *p* < 0.001). **b** Relationship of the change in GES over 1 year to the index GES value is shown. For the same 173 patients, the average GES between index and second-draw measurements increased from 15.9 to 17.3, but there was no dependence of this change on the index GES value (*r* < 0.01, *p* = NS). **c** Relationship of the change in GES between index and second GES measurements on index maximum percent stenosis is shown. There was no significant dependence of the change in GES on index stenosis (*r* < 0.01, *p* = NS)

The above results demonstrate sample and GES analytical stability but not the extent of biological variation over time on a per patient basis. To address this question, a subset of patients from the COMPASS study, who had been referred for myocardial perfusion imaging for suspected CAD, were re-consented. A second blood sample was obtained approximately 1 year after the index blood samples, which formed the basis of the previous results (Fig. 2). A pre-specified GES threshold of 15 was derived from the PREDICT validation results and validated in COMPASS with a sensitivity of 89 % and negative predictive value of 96 % [8]. Demographics for the complete COMPASS set of 431 patients for whom MPI, invasive angiography or CTA, and GES were obtained and the 195 patient subset for whom second blood samples were obtained are shown in Table 4. The mean age was 57 years, 49 % female; GES was obtained on 192 (98 %). Of these, 19 patients were censored due to revascularizations [17] and events [2] between index and 1-year sampling. For the remaining 173, the index GES was correlated with maximum percent stenosis (Fig. 3a), as was seen in the entire cohort. Between the index and second blood samples, mean scores increased from 15.9 to 17.3, corresponding to a 2.5 % increase in obstructive CAD likelihood by logistic regression, with approximately half of the increase due to increased patient age. The change in GES between patient samples was independent of index GES (Fig. 3b) and maximum percent stenosis at study entry (Fig. 3c). For those patients with revascularizations or events, the average score change was 1.1, similar to that for those without (Supplementary Table 1).

**Table 4:** Clinical and demographic characteristics of COMPASS cohorts

Clinical variable	Overall study *N* = 431^a^	Top sites *N* = 295^b^	Enrolled patients *N* = 192^c^
% obstructive CAD—core lab^d^	0.15	0.15	0.17
Male	225 (52 %)	159 (54 %)	95 (50 %)
Race—White	383 (89 %)	273 (93 %)	179 (93 %)
**Age (years)** ^e^	56 +/− 10	55 +/− 10	57 +/− 10
**Systolic BP (mmHg)**	130 +/− 17	131 +/− 18	131 +/− 18
**Dyslipidemia**	236 (55 %)	163 (55 %)	112 (58 %)
BMI	30 +/− 6	30 +/− 6	30 +/− 6
**Smoker**			
Current	66 (15.3 %)	45 (15.3 %)	24 (12.5 %)
**Aspirin**	212 (49 %)	150 (51 %)	104 (54 %)
**Beta-blockers**	86 (20 %)	66 (22 %)	42 (22 %)
ACE inhibitors	130 (30 %)	89 (30 %)	62 (32 %)

^a^The set of patients analyzed in Thomas et al. [8] for whom core-lab QCA or CTA, MPI, and GES were obtained

^b^The top four enrolling sites in the COMPASS study representing 68 % of subjects

^c^Patients from the top four sites who consented to the second blood draw and for whom a second GES was obtained (192/195 = 98 %)

^d^Obstructive CAD was defined as ≥50 % stenosis by either QCA or core-lab CTA as described in Thomas et al. [8]

^e^Clinical or demographic factors which differed between obstructive CAD cases and controls are indicated in bold face

A total of 12 patients (7 %) had scores that crossed the GES threshold of 15 between index and 1-year samples, nine increased and three decreased, none of whom had obstructive CAD at baseline. Only four patients (2 %) had score changes >10. The number of cardiovascular medications (for dyslipidemia and hypertension) prescribed during the study period increased in 29 patients without a significant change in their GES (change in score 0.7 vs. 1.5, *p* = 0.25).

## Discussion

There are four major conclusions from this study about the analytical and clinical validity of this gene expression score for obstructive CAD: (1) Expansion of the PREDICT validation cohort by almost 1,000 patients to over 1,500 subjects with clinical angiographic reads yielded the same test performance as in the original report. (2) In this significantly larger clinical read population, subjects of non-White ethnicity (*N* = 138) showed significant and similar performance as Whites (ROC AUC = 0.72 ± 0.06, *p* < 0.001). (3) The RT-PCR platform for the GES was analytically robust yielding the same performance in 501 subjects from the original validation study with little change in score over a 5-year period. (4) To examine the biological variation in GES over time, a second blood sample was obtained 1-year post-enrollment, from patients in the COMPASS study, and showed overall a small change in GES and little effect due to changes in cardiovascular medications.

The expanded results from the PREDICT study both replicated prior data and added to them by examining clinical angiographic reads as well as QCA. Other work has shown that QCA is more conservative than clinical reads [17], and our results show perhaps an even larger difference in stenosis measurement between these methods in this larger cohort (*N* = 1,038). A further analysis of these results using multivariate methods could prove interesting. Prior work on the analytical performance of this GES showed that the overall 95 % confidence intervals were approximately 2 units from the mean [15]. In this work, we show that 78 % of patients fell within this range with re-testing after 5 years of sample storage. These results suggest that PAXgene tube blood RNA stability is longer than what has been shown previously [18].

It has been suggested that given differences in both the prevalence of CAD [12] and the relative proportions of calcified plaque in different ethnicities [19] that further studies on this GES were needed. The current work suggests that the overall performance of the GES is similar in different ethnicities, although further work in larger cohorts, enabling multivariate analyses in these populations, would be desirable. With respect to plaque composition, since the GES is sensitive to both calcium score and overall plaque burden [14], the changes in the proportion of calcified plaque in different ethnicities may not have a large influence on the score.

The results from the sequential draws in the COMPASS study population show overall small changes over a 1-year time frame. Given the demonstrated quantitative relationship between the GES and atherosclerotic plaque burden, measured cross sectionally in both the PREDICT and COMPASS studies [7, 8, 14], these results suggest that disease progression is not a major factor over this time frame. Results from sequential CT-angiographic studies also show relatively modest disease progression over 1 to 2 years, although this may be confounded by medication effects on plaque progression and composition [20, 21]. Finally, there was little effect of medication changes on the GES, although the patient numbers analyzed are relatively modest. Given that the GES was derived from a mixed population with respect to medication use, it is unlikely that highly medication-sensitive genes would have been selected as CAD classifiers. A recent study examining intensive cardiovascular risk reduction in a post-MI population with sequential microarray analyses also showed little effect of medications on gene expression [22]. Specifically with respect to the GES, only one gene present in the algorithm (S100A12) was shown to be significantly changed in this risk reduction study [22].

These current studies of the GES have limitations. First, they are restricted to patients without known CAD or previous MI who are non-diabetic and do not have chronic inflammatory diseases. Second, the PREDICT population is at higher risk than the clinical population seen on suspicion of CAD in primary care or cardiology. It is subject to referral bias, since subjects were already referred for invasive angiography, and is also biased towards females in the largest cohorts. However, previously reported results in the COMPASS study suggest that test performance is relatively insensitive to disease prevalence [8], and in current and previous work, GES performance is similar for males and females [23]. Third, not all of the PREDICT patients have QCA as an anatomical gold standard, although GES performance appears to be consistent between QCA and clinical reads, when the difference in stenosis observed by the two methods is taken into account. Fourth, the COMPASS second blood draw results lack paired second anatomical measurements, which could have been useful to inform upon the small number of patients with significant score changes, and the PREDICT populations did not have a second blood draw. Lastly, although the GES provides a stable and reproducible measure of obstructive CAD likelihood, with reasonable overall diagnostic accuracy [14], the additional classification power which might be provided by other existing biomarkers or additional genomic modalities, such as epigenetics, common genetic polymorphisms, circulating micro-RNAs, and others, was not investigated and is unknown. In addition, gene discovery by next-generation RNA sequencing may identify additional classifiers as has been shown in breast cancer [24].

In summary, we have expanded the original PREDICT GES validation cohort to more than 1,500 patients and demonstrated very similar and significant performance in White and non-White ethnicities in this cohort, with sample score stability over a 5-year period also demonstrated on the original validation samples. The biological variation of the GES over time was tested by examining second-draw samples from a subset of  COMPASS study patients approximately 1-year post-study enrollment with little or no change in GES over this time period in the vast majority of patients and no significant effect of changes in cardiovascular medications.

## Electronic supplementary material

Below is the link to the electronic supplementary material.

ESM 1 (DOCX 111 kb)

ESM 2 (DOCX 108 kb)

## Abbreviations

GES: Gene expression score
CAD: Coronary artery disease
QCA: Quantitative coronary angiography
ROC: Receiver-operating characteristics
AUC: Area under the curve
CTA: Computed tomographic angiography
