# Supplementary material for: Biological and Analytical Stability of a Peripheral Blood Gene Expression Score for Obstructive Coronary Artery Disease in the PREDICT and COMPASS Studies
Source: J Cardiovasc Transl Res. 2014 Aug 14;7(7):615–22. doi: 10.1007/s12265-014-9583-3 (PMC4185104; doi:10.1007/s12265-014-9583-3)
Supplement: Supplementary file 1 — (DOCX 111 kb) [file 12265_2014_9583_MOESM1_ESM.docx]

**Gene Expression Score composite Genes and Calculation**

The GES algorithm comprises the gene expression levels of 23 genes, measured by quantitative RT-PCR, in 6 correlated terms with distinct weighting for men and women as well as sex-specific age dependent obstructive CAD likelihood functions. To determine the gene expression score for a patient, the median Cp values from RT-PCR were used for subsequent calculations, as described below. Gene symbols refer to the following: The genes symbols are: IL18RAP = IL18 receptor associated protein, TNFAIP6 = TNF-alpha induced protein 6, CASP5 = caspase-5, IL8RB = IL8 receptor beta, TNFRSF10C = TRAIL decoy receptor 3, TLR4 = toll-like receptor-4, KCNE3 = ISK family potassium voltage-gated channel, S100A8 = S100 Calcium Binding protein 8, S100A12 = S100 Calcium Binding protein 12, CLEC4e = C-type lectin domain family 4e, RPL28 = Ribosomal protein 28 light subunit, AQP9 = Aquaporin 9, NCF4 = Neutrophil cytosolic factor 4, SLAMF7 = SLAM family member 7, KLRC4 = Killer cell lectin receptor family C4, TMC8 = Transmembrane channel-like-8, CD3D = CD3-delta, SPIB = Spi-B transcription factor, CD79B = Immunoglobulin associated CD79B, AF2 = AF289562, unknown protein, TSPAN = AF161365, unknown protein, TFCP2 = Transcription factor CP2, HNRPF = Heterogeneous nuclear riboprotein F.

**Algorithm Calculation**.

The raw gene expression score is calculated from median Cp values as follows:

Raw Score = INTERCEPT – 0.755 *( N_up_ - N_down_) – 0.308 *SEX*( SCA_1_- Norm_1_) - 0.548 *(1-SEX)*( SCA_1_- Neut) – 0.406*( NK_up_ - T_cell_) - 0.137* ( B_cell_- T_cell_) - 0.482 *SEX*(TSPAN)- 0.246 ( AF2- Norm_2_)

For Males (SEX=1) and Females (SEX=0), INTERCEPT = 2.672+0.0449*Age and 1.821+0.123*(Age-60), respectively, with only positive values allowed for females;

1. Define Norm_1_ = RPL28
2. Define Norm_2_ = (.5*HNRPF + .5*TFCP2)
3. Define NK_up_ = (.5*SLAMF7 + .5*KLRC4)
4. Define T_cell_ = (.5*CD3D + .5*TMC8)
5. Define B_cell_ = (2/3 *CD79B + 1/3 * SPIB)
6. Define Neut = (.5*AQP9 + .5*NCF4)
7. Define N_up_ = (1/3 * CASP5 + 1/3*IL18RAP + 1/3*TNFAIP6)
8. Define N_down_ = (.25*IL8RB + .25*TNFRSF10C + .25*TLR4 + .25*KCNE3)
9. Define SCA_1_ = (1/3*S100A12 + 1/3*CLEC4E + 1/3*S100A8)
10. Define AF_2_ = AF289562
11. Define TSPAN = 1 if (AF161365-Norm2 > 6.27 or AF161365=NoCall), 0 otherwise
12. Define SEX= 1 for Males, 0 for Females
13. Define Intercept
    1. For Males, INTERCEPT = 2.672 + 0.0449*Age
    2. For Females, INTERCEPT = 1.821 + 0.123*(Age-60), if negative set to 0

Define Score = INTERCEPT – 0.755 *( N_up_ - N_down_) – 0.308 *SEX*( SCA_1_- Norm_1_) - 0.548 *(1-SEX)*( SCA_1_- Neut) – 0.406*( NK_up_ - T_cell_) - 0.137* ( B_cell_- T_cell_) - 0.482 *SEX*(TSPAN)- 0.246 ( AF2- Norm_2_),

1. **Score Transformation**

The endpoint analyses defined were performed using raw algorithm scores. For clinical reporting purposes, as well as ease of presentation, raw scores were transformed into a transformed score with a scale from 1-40 designed for ease of clinical use as follows:

Input is Raw Score

If Raw Score< -2.95, set RawScore = -2.95

If Raw Score> 1.57, set RawScore = 1.57

Raw Score = 2.95 + RawScore

Final Score = RawScore*40/4.52

Round Final Score up to nearest integer

If Final Score is greater than 40, set to 40

If Final Score is less than 1, set to 1

Value obtained is the Final Transformed Score
