# Supplementary material for: Biological and Analytical Stability of a Peripheral Blood Gene Expression Score for Obstructive Coronary Artery Disease in the PREDICT and COMPASS Studies
Source: J Cardiovasc Transl Res. 2014 Aug 14;7(7):615–22. doi: 10.1007/s12265-014-9583-3 (PMC4185104; doi:10.1007/s12265-014-9583-3)
Supplement: Supplementary file 2 — (DOCX 108 kb) [file 12265_2014_9583_MOESM2_ESM.docx]

Supplementary Table 1. Characteristics of COMPASS Patients who had Revascularizations or Events between Index and One Year Sampling.

| Patient ID | Age | Sex | Baseline GES | 1 Year GES | GES Change | Revasc or Event^a^ | Max Percent Stenosis^b^ | Site MPI^c^ | Core MPI^d^ |
| --- | --- | --- | --- | --- | --- | --- | --- | --- | --- |
| C101:70028 | 55 | M | 20 | 22 | 2 | Event | 0 | Normal | Abnormal |
| C113:70010 | 64 | M | 30 | 31 | 1 | Event | 60 | Normal | Normal |
| C101:70011 | 58 | M | 24 | 26 | 2 | Revasc. | 72.5 | Abnormal | Abnormal |
| C101:70043 | 52 | M | 23 | 23 | 0 | Revasc. | 85 | Abnormal | Abnormal |
| C101:70047 | 69 | M | 28 | 32 | 4 | Revasc. | 33 | Normal | Abnormal w Artifact |
| C101:70069 | 85 | M | 23 | 24 | 1 | Revasc. | 95 | Normal | Abnormal w Artifact |
| C102:70014 | 76 | M | 28 | 29 | 1 | Revasc. | 92 | Normal | Normal |
| C102:70066 | 59 | M | 23 | 27 | 4 | Revasc. | 77 | Normal | Normal |
| C102:70130 | 57 | M | 30 | 29 | -1 | Revasc. | 75 | Abnormal | Abnormal |
| C102:70157 | 49 | F | 10 | 8 | -2 | Revasc. | 78 | Abnormal | Abnormal w Artifact |
| C102:70174 | 56 | M | 25 | 20 | -5 | Revasc. | 25 | Normal | Normal |
| C106:70009 | 45 | M | 23 | 26 | 3 | Revasc. | 64 | Normal | Normal |
| C106:70019 | 58 | M | 29 | 31 | 2 | Revasc. | 100 | Normal | Abnormal |
| C113:70003 | 60 | M | 28 | 25 | -3 | Revasc. | 67 | Normal | Normal w Artifact |
| C113:70011 | 53 | M | 27 | 36 | 9 | Revasc. | 66 | Abnormal | Normal w Artifact |
| C113:70024 | 61 | F | 17 | 16 | -1 | Revasc. | 71 | Normal | Normal |
| C113:70035 | 48 | M | 27 | 31 | 4 | Revasc. | 78 | Normal | Normal |
| C113:70049 | 64 | F | 20 | 19 | -1 | Revasc. | 51 | Abnormal | Abnormal |
| C113:70051 | 71 | F | 22 | 23 | 1 | Revasc. | 71 | Normal | Normal w Artifact |

^a^ Revascularizations (PCI or CABG) or events (non-fatal MI, stroke, all-cause mortality) occurring between index GES and second blood draw.

^b^ Maximum percent stenosis determined by core-laboratory CTA or QCA at time of index GES as described[^8^](#_ENREF_8)

^c^  Myocardial perfusion imaging results from the individual site clinical reads.

^d^  MPI results from the core-laboratory reads as described[^8^](#_ENREF_8)
